# Intravesicular administration of sodium hyaluronate ameliorates the inflammation and cell proliferation of cystitis cystica et glandularis involving interleukin-6/JAK2/Stat3 signaling pathway

**DOI:** 10.1038/s41598-017-16088-9

**Published:** 2017-11-21

**Authors:** Yongliang Ni, Shaohua Zhao, Xiaoxuan Yin, Haixin Wang, Qianqian Guang, Guangxia Hu, Yi Yang, Shoubin Jiao, Benkang Shi

**Affiliations:** 1 Department of Urology, Shandong University Qilu Hospital, Jinan, Shandong 250012 China; 2 Department of Geriatrics, Shandong University Qilu Hospital, Jinan, Shandong 250012 China; 3 Department of Traditional Chinese Medicine, Yankuang Group General Hospital, Zoucheng, Shandong 273500 China; 4 Department of Urology, Yankuang Group General Hospital, Zoucheng, Shandong 273500 China; 5 Department of Pathology, Yankuang Group General Hospital, Zoucheng, Shandong 273500 China

## Abstract

Cystitis cystica et glandularis (CCEG) is a chronic cystitis that causes extreme agony in affected patients. However, there are lack of effective conservative treatments. In this study, it is evident that intravesicular sodium hyaluronate (SH) therapy significantly improved the clinical symptoms of CCEG patients and ameliorated the bladder mucosal inflammation and cell proliferation characteristics of the disease. Immunohistochemical staining showed that the staining intensities of hyaluronidase (HYAL 1/2), CD44, IL-6 and phosphorylated signal transducer and activator of transcription 3 (p-Stat3) in bladder mucosal tissue were significantly increased in CCEG patients compared with control patients and that intravesicular SH treatment suppressed these protein expression. We established a CCEG rat model by treating rats with *E*. *coli* intravesicularly, and we found that HYAL 1/2 and CD44 expression levels were significantly increased in the *E*. *coli* group compared with the NC group. Activation of the IL-6/JAK2/Stat3 pathway and the expression levels of the downstream pro-apoptotic proteins Mcl-1 and Bcl-xL were also significantly increased in the *E*. *coli* group compared with the NC group. The above changes were significantly mitigated by intravesicular SH treatment. Therefore, SH may serve as an effective therapy for CCEG by inhibiting bladder mucosal inflammation and proliferation.

## Introduction

Cystitis cystica et glandularis (CCEG) is a chronic reactive inflammatory disorder thought to be attributable to chronic urothelial irritation caused by infections, tumors, calculi or outlet obstructions^1^. CCEG is characterized by pathologic proliferative changes in the bladder mucosa. Early studies suggest that CCEG is a precancerous lesion^2,3^. Recently, more and more evidences suggest that there is no significant causal relationship between CCEG and bladder malignancies, but there exists phenomenon of coexistence of cystitis glandularis and bladder carcinoma with high ratio^4–6^.

The typical symptoms of CCEG are urinary frequency, urinary urgency, dysuria, and hematuria, which cause extreme discomfort in affected patients and reduce their quality of life. Improvements in cystoscopy and biopsy techniques have led to a gradual increase in the number of reports about CCEG over the last decade. However, the pathogenesis of CCEG remains unclear^7^. Given that CCEG is a chronic reactive inflammatory disorder that causes pathologic proliferative changes in the bladder mucosa, we elected to focus on the mechanisms responsible for the inflammation and cell proliferation characteristic of CCEG in this study.

Increasing amounts of evidence have indicated that inflammatory signals play an important role in sustaining and promoting neoplastic growth. The pro-inflammatory cytokine IL-6 and its downstream effectors, Janus-activated kinases (JAK2) and signal transducer and activator of transcription 3 (Stat3), have been demonstrated to play important roles in blocking cell apoptosis and enhancing cell proliferation in various hyperplastic diseases^8,9^. For example, IL-6 has been reported to induce phosphorylation of Stat3, which is associated with increases in the expression levels of the anti-apoptotic genes Bcl-xL and Mcl-1 in Barrett’s esophagus, a condition that appears to result from chronic irritation and is characterized by the replacement of dysplastic squamous epithelial cells with metaplastic intestinal-like columnar epithelial cells^10,11^. Similar pathologic changes occur in the bladder in CCEG. The IL-6/JAK2/Stat3 pathway plays a critical role in inflammation and proliferation. Both of inflammation and proliferation occur in the bladder mucosa in CCEG. Thus, it is hypothesised that the IL-6/JAK2/Stat3 pathway is also involved in CEGG development and progression. Thus, in this study, we investigated the activity of the IL-6/JAK2/Stat3 signaling pathway and assessed the expression of the downstream anti-apoptotic biomarkers Mcl-1 and Bcl-xL to elucidate the molecular mechanisms underlying the development of CCEG.

Glycosaminoglycans (GAGs) form a thick layer that covers the bladder epithelium to block various irritants, such as chemicals, calculi and bacteria that cause chronic infections^12^. The protection provided by GAG layers may prevent the constant evolution of bladder inflammation. GAGs have recently become a novel therapy for the treatment of recurrent urinary tract infections and interstitial cystitis/painful bladder syndrome (IC/PBS)^13–15^. Endogenous hyaluronic acid (HA) is a key component of GAGs, and recent studies have suggested that intravesicular instillation of sodium hyaluronate (SH) may promote regeneration of the GAG layers on the bladder urothelium and inhibit IL-6 secretion in bladder tissue^16^. However, few studies have investigated the effects of SH treatment on human CCEG. Thus, in the present study, we evaluated the effects of treatment with SH on CCEG patients and elucidated the mechanisms linking the IL-6/JAK2/Stat3 pathway to CCEG in a CCEG rat model.

## Results

### SH ameliorated bladder mucosal inflammation and cell proliferation and thus improved the clinical symptoms of CCEG patients

All CCEG patients were given pre- and post-treatment “PUF Patient Symptom Scale Questionnaires” to assess the effects of treatment with SH on their clinical symptoms. The voiding dairies completed by the patients enrolled herein were used to estimate their mean daytime urinary frequency and their maximum bladder volume. After SH treatment, CCEG patients displayed significant improvements in their bladder pain, daytime urinary frequency and maximum bladder volumes (Table 1). With the exception of one patient who experienced two episodes of transient whole-body itching, no patients experienced severe adverse events during the indicated period.

**Table 1:** Severity of patient symptoms and inflammation before and after SH treatment.

Point-in-time	PUF score	frequency	MBC	Histological score	inflammatory cells counts	Brunn’s nests counts
Pre-treatment	12.95 ± 2.11	18.15 ± 2.43	221.57 ± 36.36	2.25 ± 0.74	77 ± 20.52	4.63 ± 1.09
Post-treatment	4.76 ± 1.28**	10.66 ± 2.17**	331.45 ± 41.62**	1.03 ± 0.48**	22.81 ± 6.58**	2.22 ± 0.42**

Values are expressed as the mean ± SD. **P < 0.01 vs. pre-treatment.

**Figure 1:**
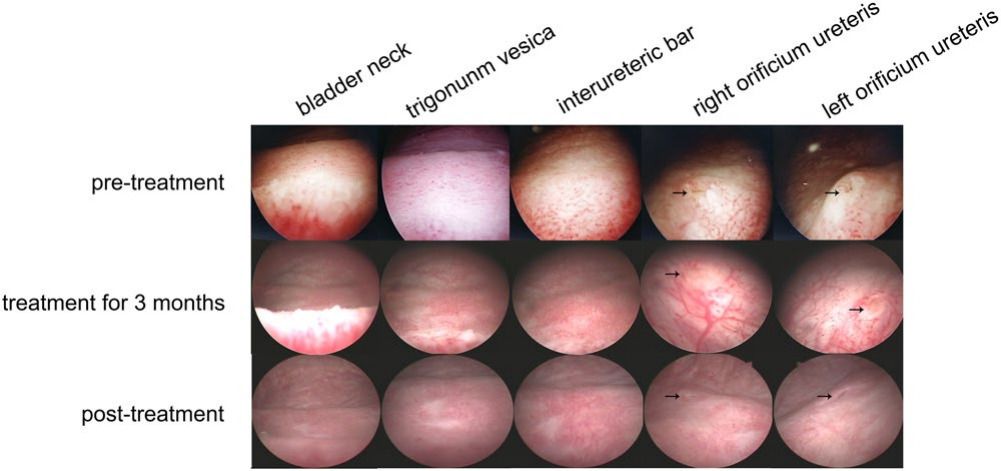
Cystoscopy showed improvement of bladder mucosal lesions in CCEG patients at different time points during SH treatment. The arrows indicate the orificium ureteris.

In the control group, the mucosa of the bladder trigonum and bladder neck appeared pink and smooth under 70° cystoscopy. In 16 CCEG patients, the bladder trigonum and bladder neck displayed white villous, follicular, papillary changes before SH treatment. Eleven of the indicated patients had inflammatory congestion. All CCEG patients experienced varying degrees of improvement in the appearance of their bladder mucosal tissues after being treated with SH for 6 months, and 6 patients displayed normal bladder mucosa under cystoscopy, i.e., mucosa that appeared pink and smooth under cystoscopy, after treatment with SH (Fig. 1).

**Figure 2:**
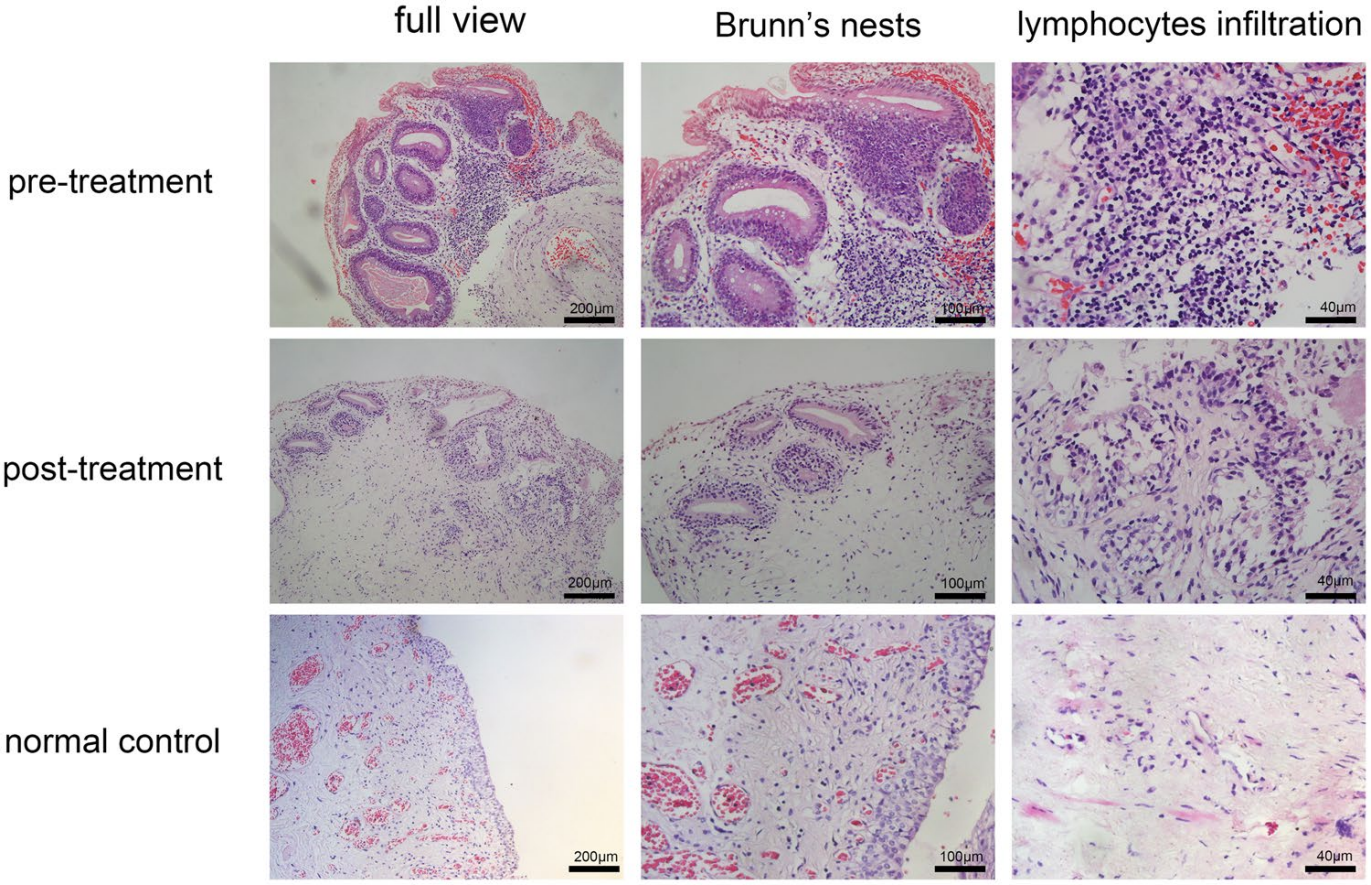
Representative histological images of HE staining showed that SH treatment ameliorated bladder mucosal inflammation and cell proliferation for CCEG patients.

HE staining showed that patients in the control group had normal bladder mucosal tissues with an intact epithelial layer and that no pathological changes had occurred, and no inflammatory cell infiltrates were present in the submucosa. Severe epithelial damage, Brunn’s nests, cysts and inflammatory cell infiltrates were observed in the submucosa in all the samples obtained from patients in the pre-treatment group. Treatment with SH significantly ameliorated CCEG-induced inflammation and cell proliferation in the bladder mucosa in the post-treatment group. The patients in this group were found to have an intact epithelial layer and decreased numbers of Brunn’s nests and cysts and less severe inflammatory cell infiltration compared with their counterparts in the pre-treatment group (Fig. 2). The data pertaining to patient histological scores, inflammatory cell counts and Brunn’s nest counts demonstrated that CCEG induced severe inflammation and significant cell proliferation in the pre-treatment group and that these changes were significantly ameliorated in the post-treatment group (Table 1).

### Immunohistochemical (IHC) analysis of HYAL 1/2, CD44, IL-6, p-Stat3, Stat3, Mcl-1 and Bcl-xL in human samples

**Figure 3:**
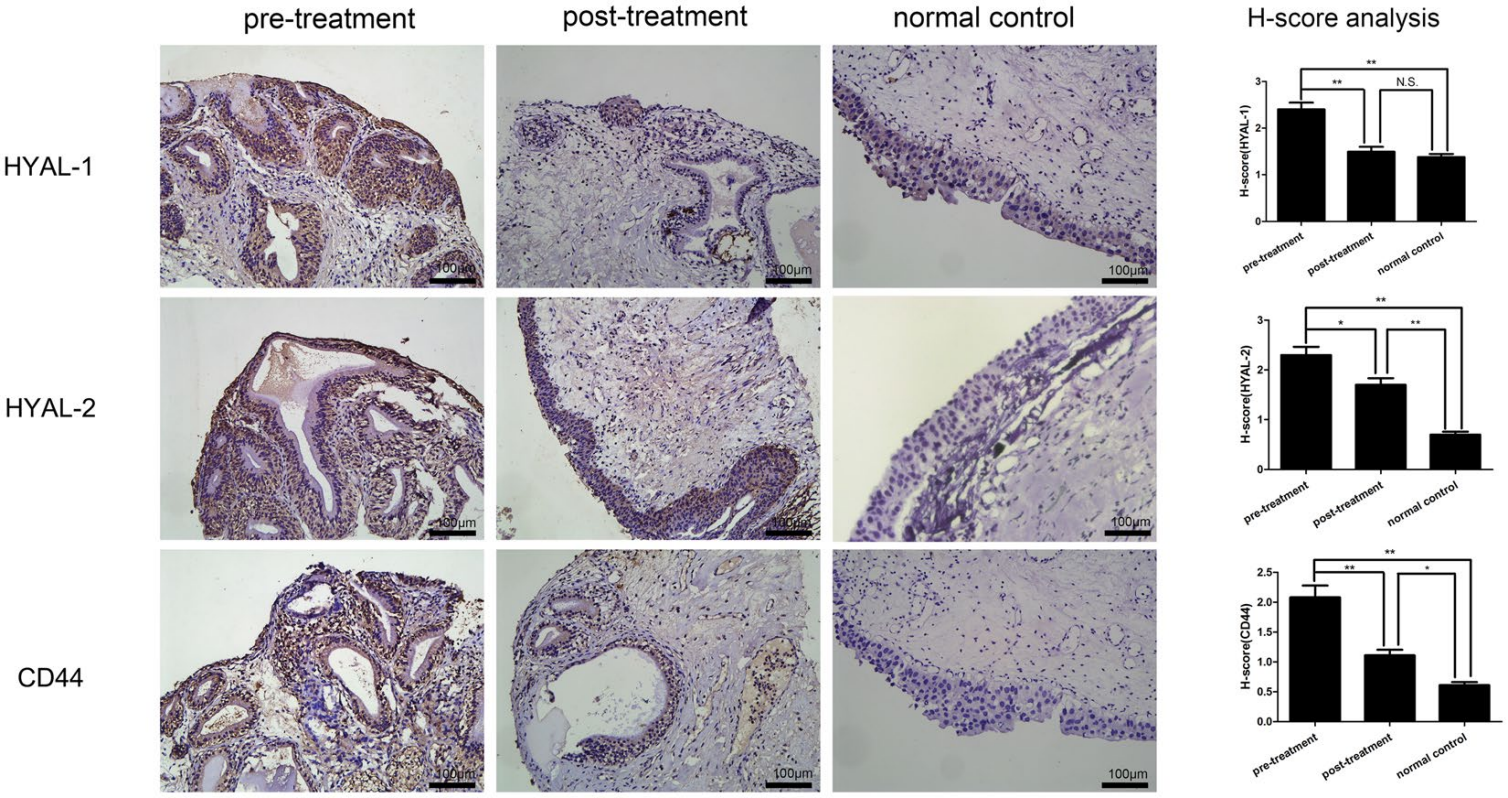
Representative images of IHC staining for HYAL-1, HYAL-2 and CD44 in the bladder mucosa (bar = 100 μm) and comparison of H-scores among the three groups. Values are expressed as mean ± SEM, n = 16 in pre-treatment group and post-treatment group, n = 6 in normal control group. *P < 0.05, **P < 0.01, N.S., non significant.

Under pathological conditions, internalized HA is degraded by hyaluronidases (such as HYAL 1/2)^17^, and the products of this degradation have been demonstrated to mediate extensive inflammatory responses by interacting with the CD44 receptor^18–20^. We detected the HYAL 1/2 and CD44 expression and localization in human samples using the semi-quantitative IHC analysis. The results showed that the HYAL 1/2 and CD44 were localized in urothelial cells and their staining intensities were significantly increased in the pre-treatment group compared with the control group. After SH treatment, the staining intensities of HYAL 1/2 and CD44 were significantly decreased in the post-treatment group (Fig. 3).

**Figure 4:**
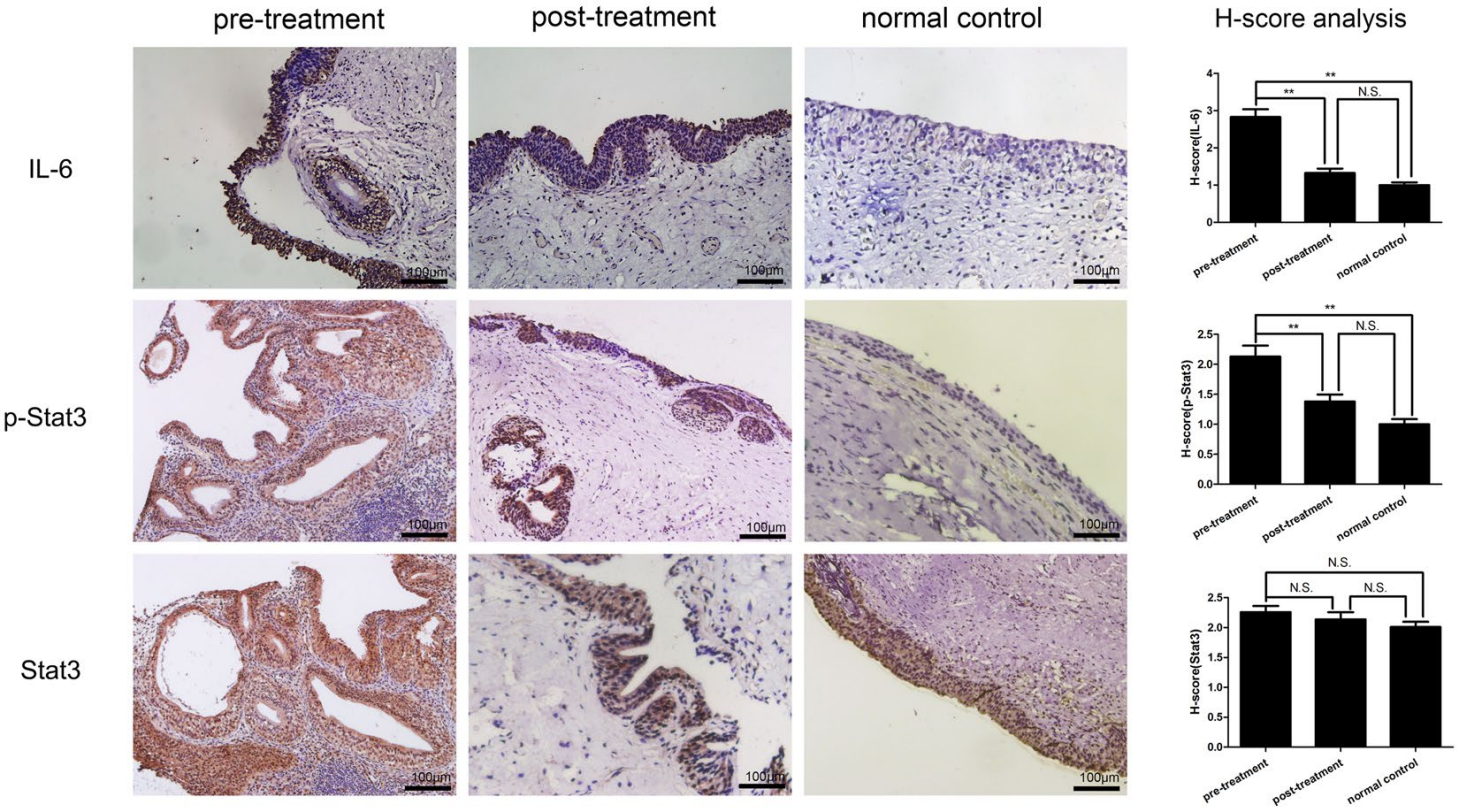
Representative images of IHC staining for IL-6, p-Stat3 and Stat3 in the bladder mucosa (bar = 100 μm) and comparison of H-scores among the three groups. Values are expressed as mean ± SEM, n = 16 in pre-treatment group and post-treatment group, n = 6 in normal control group. *P < 0.05, **P < 0.01, N.S., non significant.

Previous researches have shown that endogenous HA fragments induced IL-6 production via a CD44-independent mechanism in bone marrow cells^21^, and IL-6 activated Stat3 pathway in various cancers^9,22,23^. Here, IHC staining was used to examine IL-6 and p-Stat3 expression and localization (Fig. 4). IL-6 and p-Stat3 were localized in urothelial cells and their staining intensities were significantly increased in the pre-treatment group compared with the control group. After SH treatment, the staining intensities of IL-6 and p-Stat3 were significantly decreased in the post-treatment group compared with the pre-treatment group. Spearman correlation coefficient test was performed to analyze the relationship between IL-6 and p-Stat3. A significant positive correlation emerged between the IL-6 H-score and p-Stat3 H-score. (R = 0.655, P = 0.006 in the pre-treatment group; R = 0.546, P = 0.029 in the post-treatment group; and R = 0.586, P = 0.017 in the H-score difference between the pre-treatment group and the post- treatment group).

The total Stat3 expression levels were also compared among pre-treatment, post-treatment and control group. Figure 4 shows that the biopsy specimens from patients in the pre-treatment, post-treatment and control groups displayed similar levels of total Stat3 expression despite of different levels of p-Stat3 expression, suggesting that increases in p-Stat3 expression are not caused by total Stat3 upregulation and that treatment with SH does not affect total Stat3 expression.

**Figure 5:**
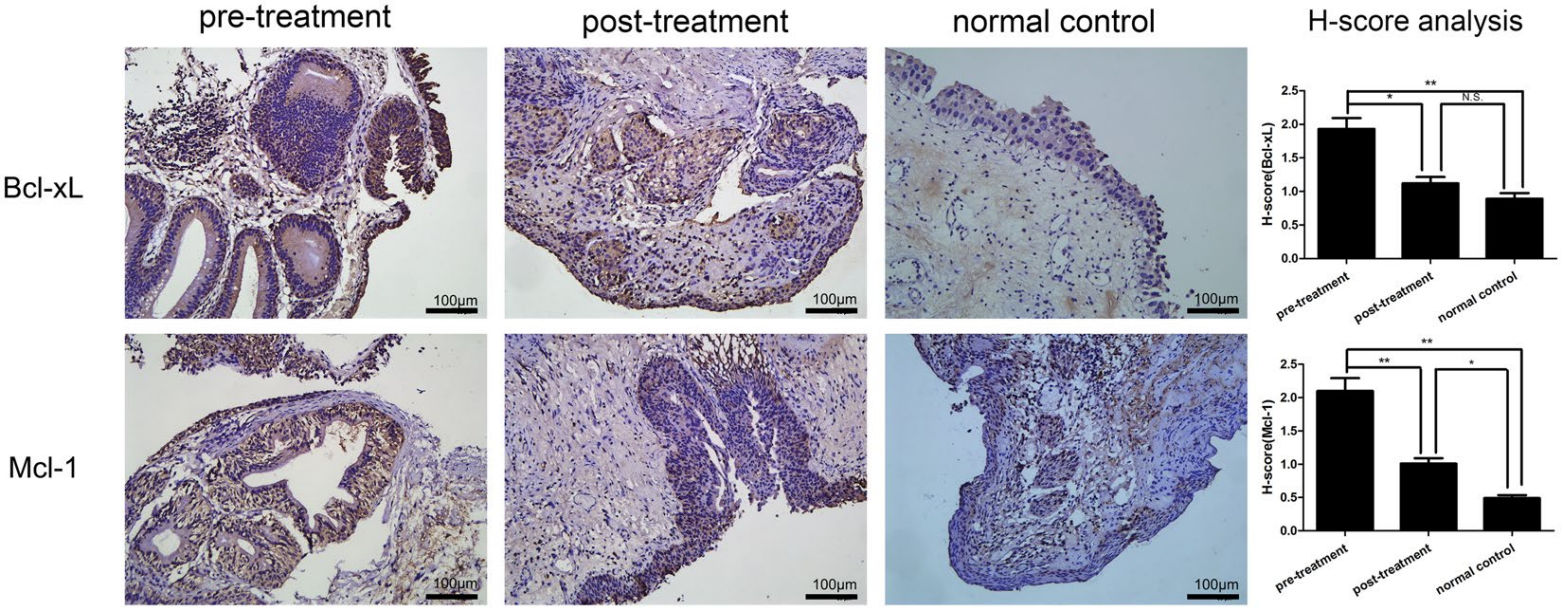
Representative images of IHC staining for Bcl-xL and Mcl-1 in the bladder mucosa (bar = 100 μm) and comparison of H-scores among the three groups. Values are expressed as mean ± SEM, n = 16 in pre-treatment group and post-treatment group, n = 6 in normal control group. *P < 0.05, **P < 0.01, N.S., non significant.

The expression and location of Bcl-xL and Mcl-1 were also detected through IHC staining analysis. Bcl-xL and Mcl-1 were mainly localized in the basal layer of urothelial cells and their staining intensities were significantly increased in CCEG patients and were significantly decreased after SH treatment (Fig. 5).

### Intravesicular administration of SH attenuated *E*. *coli*-induced cystitis in rats

The genesis of CCEG has been shown to be strongly correlated with chronic lower urinary tract infections, particularly those caused by *E*. *coli* ^7^. Intravesicular administration of *E*. *coli* has been reported to induce cystitis glandularis in rats^24^. Here, we used a similar method to generate an *E*. *coli*-induced CCEG rat model, whose successful establishment was subsequently confirmed by HE staining. The bladder mucosa in the NC group was normal and consisted of 4-6 layers of epithelial cells of an identical size. No heterocysts were present, and no inflammatory cellular infiltrates were observed in the submucosal layer. Brunn’s nests, cystitis cystica and cystitis glandularis were observed in 12 samples in the *E*. *coli* group. The glands were covered with the transitional epithelium of the urinary tract, and significant inflammatory cell infiltration was noted in the vicinity of the Brunn’s nests and cysts.

**Figure 6:**
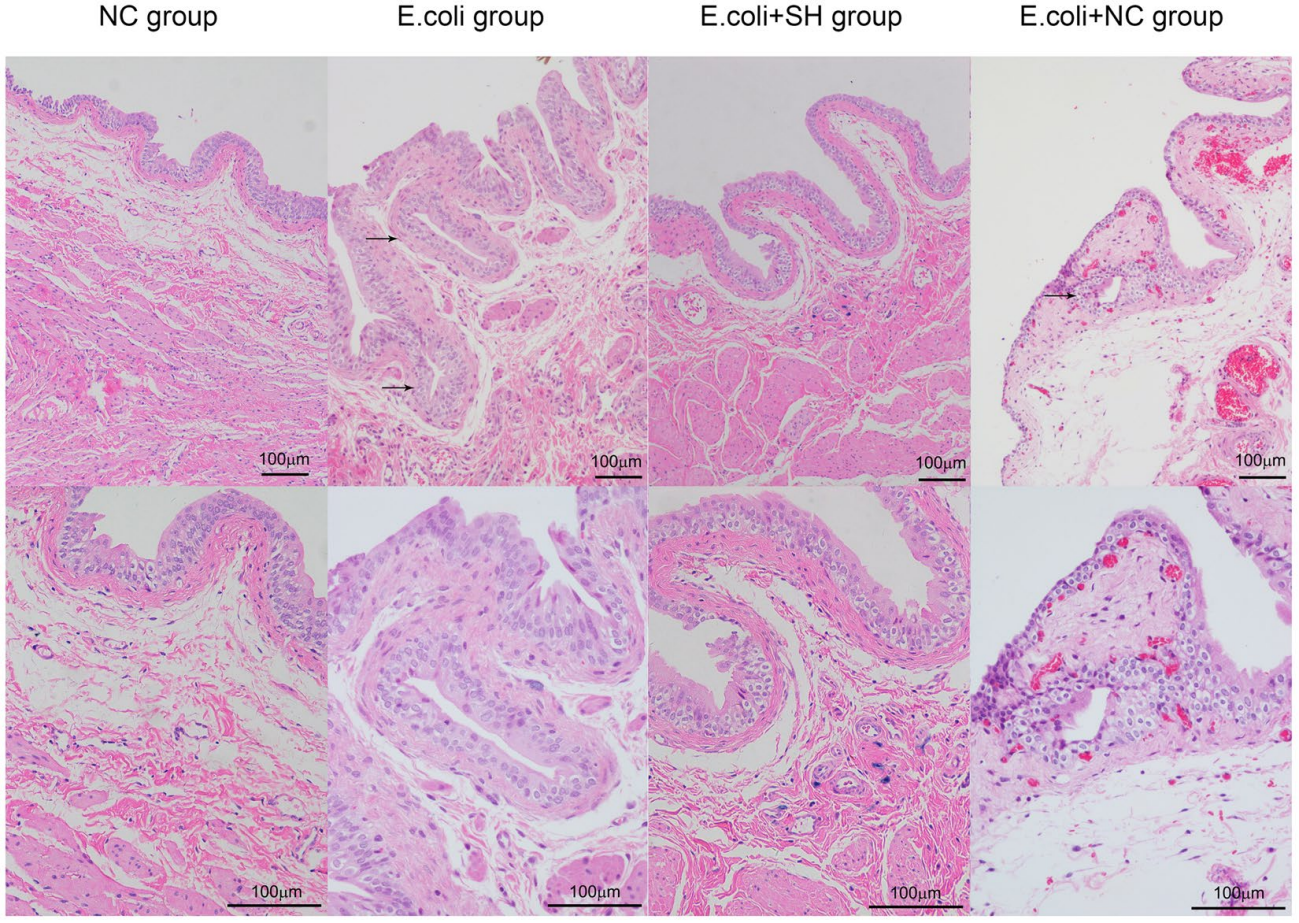
Representative HE staining images of rat bladder in each group. The arrows indicate Brunn’s nests (bar = 100 μm).

Intravesicular administration of HA can improve bacterial cystitis and attenuate cystitis-induced hypercontractility in rats^25^. Here, we evaluated the effects of SH in rats with CCEG. The histopathologic changes observed in the *E*. *coli* + NS group were similar to those observed in the *E*. *coli* group; however, inflammatory cell infiltration was less severe in the former group than in the latter group. Moreover, Brunn’s nest and cyst numbers were significantly decreased in the *E*. *coli* + SH group compared with the *E*. *coli* + NS group (Table 2). Additionally, no significant inflammatory cell infiltrates were noted in the vicinity of the Brunn’s nests and cysts in the *E*. *coli* + SH group (Fig. 6).

**Table 2:** Histological evaluation in rat *E. coli*-induced cystitis.

Group	NC group	*E. coli* group	*E. coli*+SH group	*E. coli*+NS group
histological score	0.24 ± 0.47	2.67 ± 0.32^a^	1.04 ± 0.35^a,b^	2.44 ± 0.64^a^
inflammatory cell counts/mm^2^	4.26 ± 2.35	100.47 ± 19.63^a^	13.54 ± 6.38 ^a,b^	70.84 ± 12.51^a^
Brunn’s nest counts/mm^2^	1.25 ± 0.61	16.67 ± 3.74^a^	4.17 ± 1.2^a,b^	12.5 ± 3.26^a^

Data was expressed as the mean ± SD, ^a^P < 0.05 vs. NC group, ^b^P < 0.05 vs. *E. coli* + NS group.

### SH treatment reduced the expression of HYAL 1/2 and CD44 in *E*. *coli*-induced cystitis glandularis rats

**Figure 7:**
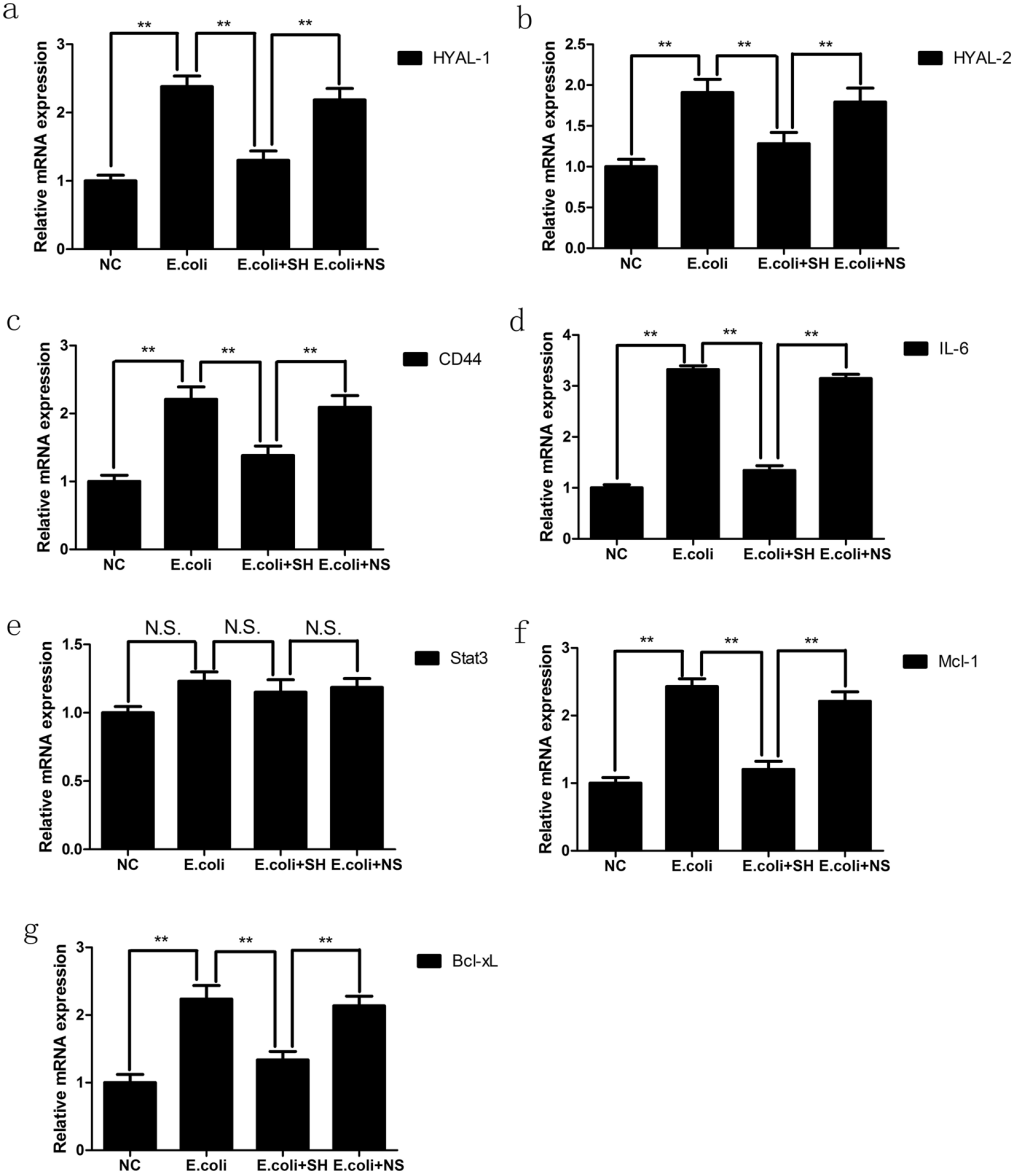
The mRNA expression of HYAL-1, HYAL-2, CD44, IL-6, Stat3, Mcl-1 and Bcl-xL in bladder mucosa in rat models, as determined by real-time PCR. GAPDH was used as an internal RNA loading control. Results are expressed as fold relative to mRNA levels in NC group (equal to 1) and represent the average value of three separate experiments (n = 20 in each group). **P < 0.01, N.S., non significant.

**Figure 8:**
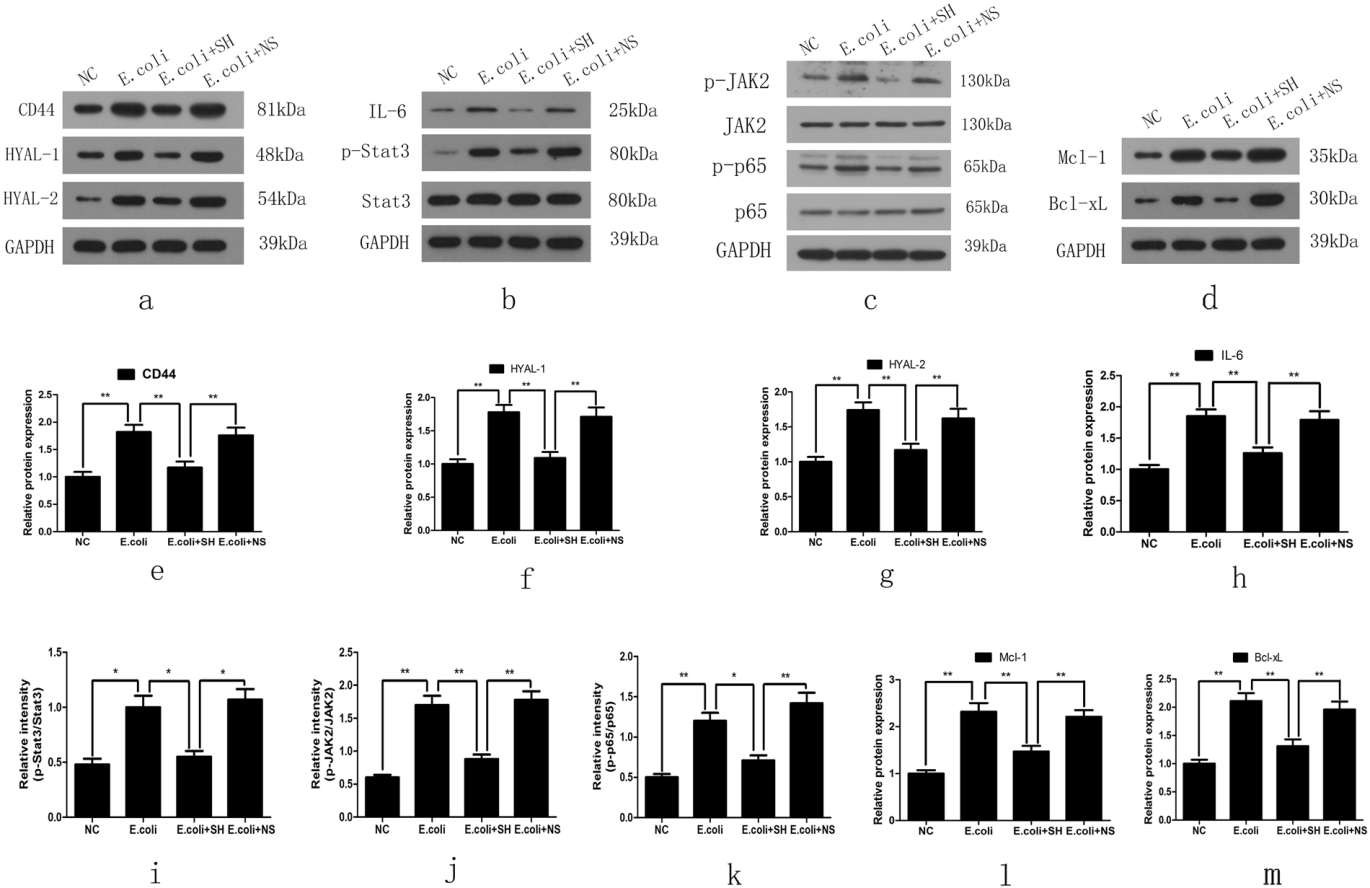
Representative blot (top panel) and densitometric analysis (low panel) of CD44, HYAL-1, HYAL-2, IL-6, Stat3, p-Stat3, JAK2, p-JAK2, p65, p-p65, Mcl-1 and Bcl-xL protein expression in bladder mucosa in rat models. Results are shown as mean ± SEM (n = 20 in each group), *P < 0.05 and **P < 0.01.

**Figure 9:**
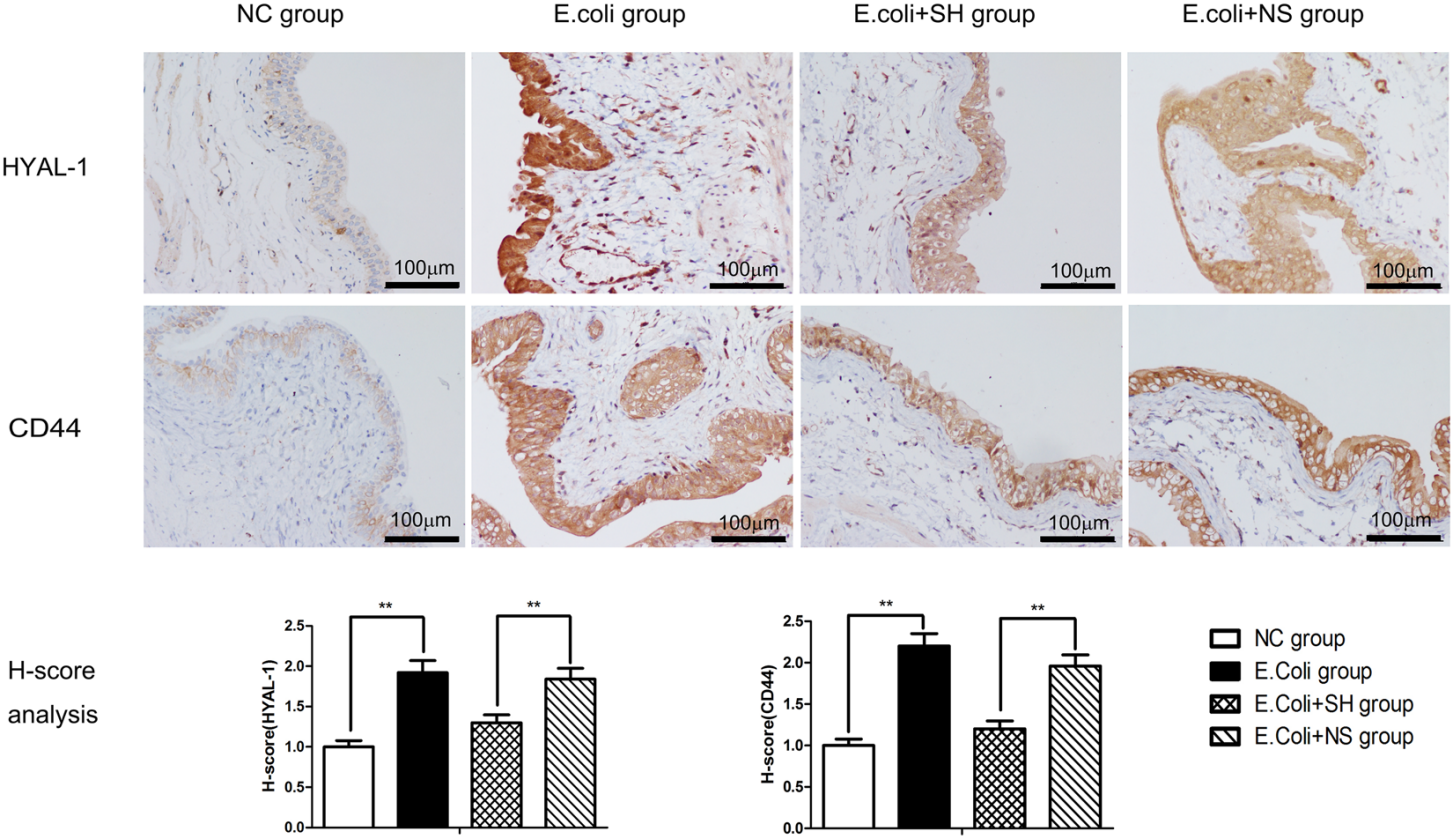
Representative images of IHC staining for HYAL-1, CD44 in the rat bladders and comparison of H-scores among the four groups (bar = 100 μm). Values are expressed as mean ± SEM, n = 20 in each group. *P < 0.05, **P < 0.01.

Real-time quantitative PCR and western blotting were performed to determine the expression levels of HYAL 1/2 and CD44 in rat bladder mucosal tissues. The results showed that bacterial infection induced increases in the mRNA and protein expression of HYAL 1/2 and CD44 in the *E*. *coli* group compared with the NC group. In contrast, their expression levels were significantly decreased in the *E*. *coli* + SH group compared with the *E*. *coli* + NS group, indicating that intravesicular administration of SH plays important roles in decreasing hyaluronidase expression and preventing internalized HA degradation (Fig. 7a–c and Fig. 8a). IHC staining was performed to detect the expression and location of HYAL1 and CD44, staining showed that HYAL1 mainly located in the cytoplasm of urothelial cells while CD44 mainly located on their membrane. Their expression trend is consistent with the expression trend of western blot (Fig. 9).

### SH treatment inhibited the activation of NF-κB and IL-6/JAK2/Stat3 pathway in E.Coli-induced cystitis glandularis rats

NF-κB is not only an important transcription factor in inflammatory responses but also a key player in anti-apoptotic signaling. We detected the NF-κB p65 activation in rat models by western blotting analysis. Results showed that p-p65 expression levels were significantly increased in the *E*. *coli* group compared with the NC group and significantly decreased in the *E*. *coli* + SH group compared with the *E*. *coli* + NS group (Fig. 8c and k).

We then evaluated the activity of the IL-6/JAK2/Stat3 pathway and the expression of its downstream anti-apoptotic proteins in rat models using real-time quantitative PCR and western blotting. The results showed that IL-6, p-JAK2 and p-Stat3 expression levels were significantly increased in the *E*. *coli* group compared with the NC group and significantly decreased in the *E*. *coli* + SH group compared with the *E*. *coli* + NS group (Figs 7 and 8). Moreover, the expression levels of the anti-apoptotic markers, Bcl-xL and Mcl-1, were significantly increased in the *E*. *coli* group compared with the NC group and significantly decreased in the *E*. *coli* + SH group compared with the *E*. *coli* + NS group (Figs 7 and 8).

## Discussion

CCEG is a common proliferative disorder of the urinary bladder whose incidence has continued to rise in recent years due to the popularity of cystoscopy^26^. CCEG patients usually suffer from recurring symptoms of chronic irritation, such as urinary frequency, urinary urgency, dysuria, suprapubic and perineal discomfort and hematuria^27^. Most patients who are admitted to the hospital for hematuria or long-term bladder irritation cannot be cured of their diseases^27^. The cause of CCEG remains ambiguous; however, the majority of investigators believe that the disease is induced by chronic inflammation or irritation^9,11^. Infection causes primary irritation of the urothelium, and long-term chronic infection may cause the transitional epithelium to proliferate into buds, invade the lamina propria and differentiate into intestinal columnar epithelial mucin-secreting glands (cystitis glandularis) or cystic deposits (cystitis cystica)^28^. CCEG is diagnosed based on the presence of characteristic pathological phenomena, such as increases in the numbers of nest-like structures known as Brunn’s nests, secretory cyst development, and lymphocyte infiltration^29^. In this study, we established a CCEG rat model via long-term intravesicular instillation of *E*. *coli* and verified the successful establishment of the model by histopathologically examining tissue specimens under a microscope after staining them with HE.

Currently, treating patients with severe CCEG entails removing the source of irritation; administering antibiotics, NSAIDs, steroid pulse therapy and anti-allergy drugs; and performing transurethral excision and fulguration of the inflamed area^30^. Oral medications usually have limited effectiveness, and surgery is too painful for many patients. Thus, new effective therapies causing only limited amounts of pain are urgently needed for the treatment of CCEG patients. Injury of the GAG layers of the bladder is a main cause of various types of cystitis. Intravesicular administration of SH, a mucopolysaccharide that constitutes an important proportion of GAGs, has been shown to have excellent efficacy with respect to the treatment of IC, radiation-induced cystitis or recurrent bacterial cystitis^31–34^. However, few studies have focused on the efficacy of intravesicular SH therapy for CCEG patients. Here, for the first time, we observed the effects of treatment with SH on bladder mucosal inflammation and cell proliferation in CCEG patients. We found that a course of intravesicular SH treatment significantly attenuated the mucosal inflammation and proliferation characteristic of CCEG in all patients with the disease. Six patients displayed normal bladder mucosa under cystoscopy, which appeared pink and smooth after SH treatment. Furthermore, HE staining revealed that Brunn’s nest and cyst counts were significantly decreased and that lymphocytic infiltration in the vicinity of Brunn’s nests and cysts was reduced in the post-treatment group compared with the pre-treatment group. Patients clinical symptoms also improved—findings supported by our observation of decreases in PUF scores, reductions in daytime urinary frequency and increases in maximum bladder volume in the post-treatment group compared with the pre-treatment group—and thus exhibited changes consistent with those noted in the experiments in which bladder mucosal histopathology was assessed.

As intravesicular SH therapy improved clinical symptoms and alleviated bladder mucosa proliferation and inflammation in CCEG patients, we investigated the molecular mechanisms underlying the effects of treatment with SH on CCEG using CCEG animal models. Endogenous HA is a major component of the extracellular matrix under normal conditions, and many studies have shown that HA is degraded into small fragments by hyaluronidases (such as HYAL1/2) when tissue injury occurs^17^. HYAL1 degrades HA into oligosaccharides, while HYAL2 degrades HA into intermediate-sized fragments (~20 kDa)^35^. CD44 is a major cell surface receptor for small HA fragments, and interactions between CD44 and HA fragments can trigger various downstream signaling pathways, including pathways associated with inflammation, proliferation, and angiogenesis induction^36^. In collagen-induced mouse arthritis, small HA fragments produced by HYALs activated CD44 and induced upregulation of the pro-inflammatory cytokine IL-6^18^. In normal human dermal fibroblasts, low-molecular-weight hyaluronan is believed to activate cells that participate in wound healing by interacting with HA receptors, such as CD44, and inducing IL-6 expression^20^. Adding small HA fragments to cultured normal chondrocytes induced severe inflammation by upregulating CD44 expression, activating NF-κB translocation and upregulating the pro-inflammatory cytokines TNF-α, IL-6 and IL-1β^37^. In this study, we found that HYAL1/2 and CD44 expression levels were significantly increased in the bladder mucosa of rats with CCEG compared with the bladder mucosa of normal control rats. These results suggested that endogenous HA was degraded into small fragments by HYAL1/2 and then interacted with CD44 in the bladder mucosal tissues of rats with CCEG. We concluded that this may be the critical mechanism by which the proliferation and inflammation characteristic of CCEG occurs. We also postulated that inhibiting endogenous HA degradation or blocking the interactions between small HA fragments and CD44 may prevent the proliferation and inflammation characteristic of CCEG. In this study, we found that intravesicular SH therapy significantly inhibited HYAL1/2 and CD44 overexpression in CCEG bladder mucosal tissues. Taken together, these results indicate that intravesicular SH therapy may inhibit the inflammatory process activated by CD44, as well as the initial process through which endogenous HA is degraded.

IL-6 is a critical cytokine in tumorigenesis, and early studies indicated that IL-6 act as a pro-tumorigenic agent in many cancers, implying that it plays an important role in proliferation^38,39^. IL-6 is also an important marker of acute or chronic inflammation. Previous studies showed that urine IL-6 levels increased in patients with IC/BPS and appeared to be correlated with symptom and inflammation severity in such patients^40,41^. Our study showed that IL-6 mRNA and protein expression levels were elevated in CCEG bladder mucosal tissues, suggesting that IL-6 plays a critical role in the inflammation and proliferation characteristic of CCEG. IL-6/JAK2/Stat3 pathway is a canonical cascade in inflammation and cell proliferation. The elevated activity of JAK2 and Stat3 is frequently observed in a variety of human malignancies^38,42,43^. We examined JAK2 and Stat3 activity levels in the indicated tissues and found that p-JAK2 and p-Stat3 expression levels were significantly elevated in the *E*. *coli* group compared with the NC group. This result was consistent with those of a previous study, which showed that p-Stat3 activation is increased in bladder epithelial cells in CCEG patients. A previous study demonstrated that intravesicular SH therapy significantly decreased cytokine IL-6 secretion, increased sulfated GAGs production and improved clinical symptoms in patients with IC^16,32^. Additionally, Stat3 signaling pathway inhibition improved bladder function after urinary bladder inflammation^44^. Thus, we speculated that intravesicular SH therapy may also serve as a treatment for CCEG by inhibiting the IL-6/JAK2/Stat3 pathway. We tested this theory in an animal model and found that IL-6, p-JAK2 and p-Stat3 expression levels were significantly decreased in the bladder mucosal tissues of the *E*. *coli* + SH group compared with those of the *E*. *coli* group after the former group received intravesicular SH therapy. The current study demonstrated that intravesicular SH therapy can suppress bladder epithelial cell proliferation and inflammation through the IL-6/JAK2/Stat3 signaling pathway.

After being activated by IL-6, Stat3 dimerizes, translocates to the nucleus, binds to gene-promoter sequences and induces the expression of specific genes^45^. Activated Stat3 regulates several genes, including the anti-apoptotic and proliferation-related genes Bcl-xL, Bcl-2, and Mcl-1^46,47^. Bcl-xL and Mcl-1 are anti-apoptotic members of the Bcl-2 family and are significantly overexpressed in bladder cancer^48,49^. Therefore, Bcl-xL and Mcl-1 are key regulators of apoptosis in bladder proliferative diseases. We evaluated Bcl-xL and Mcl-1 expression in the bladder tissues of rats with CCEG. The results showed that Bcl-xL and Mcl-1 expression was significantly increased in the *E*. *coli* group compared with the NC group and that intravesicular SH therapy significantly suppressed Bcl-xL and Mcl-1 expression in the corresponding group compared with the *E*. *coli* group. Given that the changes in Bcl-xL and Mcl-1 expression levels were consistent with the activation of IL-6/JAK2/Stat3 signaling pathway, we speculated that an important mechanism underlying the upregulation of Mcl-1 and Bcl-xL is the IL-6/JAK2/Stat3 signaling pathway.

Previous studies have shown that inducing the expression of the anti-apoptotic proteins in the Bcl-2 family promoted resistance to cisplatin-induced apoptosis by activating the NF-κB pathway in bladder cancer cells and tissues^49,50^. In this study, the activity of NF-κB p65 were significantly increased in the *E*. *coli* group compared with the NC group and significantly decreased after SH treatment. NF-κB is not only an important transcription factor in inflammatory responses but also a key player in anti-apoptotic signaling. This further confirmed that SH treatment can significantly improve the inflammation and proliferation of bladder mucosa in CCEG rats.

## Materials and Methods

### Antibodies and reagents

Antibodies specific for CD44, HYAL-1, HYAL-2, IL-6, Stat3, phospho-Stat3, Mcl-1, Bcl-xL and GAPDH were obtained from Abcam (Cambridge, UK), antibodies to JAK2, phosphor-JAK2, p65, phosphor-p65 were purchased from Cell Signaling Biotechnology (Hertfordshire, England) and sodium hyaluronate (SH; Cystistat®) was purchased from Mylan Institutional (Coill Rua, Inverin, County Galway, Republic of Ireland).

### Diagnosis of CCEG and intravesicular SH therapy

Of all the outpatients admitted to the Department of Urology, Yankuang Group General Hospital, Zoucheng, China, between Mar 2013 and Jun 2016, 16 were histopathologically diagnosed with typical CCEG. The study protocol was approved by the Institutional Review Board of Yankuang Group General Hospital, Zoucheng, China, and written informed consent was obtained from each patient who participated in the study. The corresponding author confirmed that all methods were performed in accordance with relevant guidelines and regulations. Following bladder catheterization, each patient received 50 ml of SH solution and then rested for 1 h. The above treatment was administered once per week for the first 12 weeks of therapy and was then administered once every two weeks for the next 3 months, after which the participants were followed up for another 3 months. Cystoscopy and biopsy were performed before and after intravesicular SH therapy.

### Evaluation of the effectiveness of intravesicular SH therapy

All the patients were given pre- and post-treatment “Pelvic Pain and Urinary/Frequency (PUF) Patient Symptom Scale Questionnaires” to assess the severity of their symptoms. Moreover, the patients were asked to keep a 3-day voiding diary pre- and post-treatment to record their maximum bladder capacity (MBC) and any episodes of urinary frequency or nocturia. Patient tissue samples were analyzed by a pathologist blinded to the patients’ clinical characteristics, and bladder histological scores were determined using a four-point scoring system.

### Human bladder specimen harvesting and classification

Sixteen bladder tissue specimens were obtained from CCEG patients (age: 42–61 years)—as diagnosed via histopathological analysis—via cystoscopic tissue biopsy by the Department of Urology, Yankuang Group General Hospital. The above bladder tissue specimens were obtained from the bladder trigonum pre- and post-treatment and were thus assigned to pre-treatment and post-treatment groups, respectively. Six normal bladder tissue specimens, which were obtained from patients undergoing transurethral bladder tumor resection, served as normal controls. All patients provided informed consent regarding the use of their samples in the study, which was approved by the Institutional Review Board of Yankuang Group General Hospital and was performed in accordance with established national and institutional ethical guidelines pertaining to the treatment of human subjects and the use of human tissue specimens for research.

### Establishment of the *E*. *coli*–induced cystitis animal model

All animal experiments complied with the ARRIVE guidelines, were performed in accordance with the National Institutes of Health Guide for the Care and Use of Laboratory Animals (NIH Publications No. 8023, revised 1978) and were approved by the Ethics Committee on the Care and Use of Laboratory Animals, Qilu Hospital, Shandong University, Jinan, P. R. China. For these experiments, 80 female Sprague–Dawley rats weighing 200–230 g were randomized into blank control (NC), Escherichia coli (*E*. *coli*), Escherichia coli + saline (*E*. *coli* + NS) and Escherichia coli + SH (*E*. *coli* + SH) groups, each of which comprised 20 rats. *E. coli* DH5-alpha was chosen and the concentration of *E. coli* was turbidimetrically determined with the concentration maintained at 5 × 10^7^/ml level. The rats in the NC group were raised under standard conditions for 2 months and did not receive any treatments. A PE-50 transurethral catheter was inserted into the bladder of each rat in the *E*. *coli* group to empty the bladder, after which each rat received an intravesicular infusion of 0.5 mL of *E*. *coli* and then rested for 30 min. The rats in the indicated group were then treated with *E*. *coli* three times a week for 2 months. The rats in the *E*. *coli* + NS group received the same pre-treatment as the rats in the *E*. *coli* group and then received intravesicular infusions of NS three times a week for 2 months. The rats in the *E*. *coli* + SH group received the same treatment as the rats in the *E*. *coli* group and then received intravesicular infusions of SH three times a week for 2 months. Within 48 h after the last treatment, all the rats were anesthetized with chloral hydrate solution for bladder tissue specimen harvesting.

### Bladder histological evaluation

The bladder tissue specimens from the patients and each group of rats were fixed in a 4% formaldehyde solution for 48 h, dehydrated in graded ethanol solutions, and then embedded in paraffin before being cut into 5-μm-thick sections. After staining the specimens with hematoxylin and eosin (HE), we performed morphological analysis of the bladder mucosa by light microscopy. We examined five random fields in each section at 40 × magnification, after which we calculated a histological score for each section and performed inflammatory cell and Brunn’s nest counts. The tissue sections were evaluated by a pathologist from the Department of Pathology, Yankuang Group General Hospital, who was blinded to the patients’ and rats’ clinical characteristics. Bladder histological scores were determined using a four-point scoring system (0, morphologically unremarkable, with no or minimal inflammatory cell inflammation or epithelial changes; 1, mild inflammatory cell infiltration in the lamina propria characterized by the presence of scattered lymphocytes or monocytes and accompanied by mild chronic edema, hemorrhage or urothelial changes; 2, moderate inflammatory cell infiltration within the lamina propria that extends into the muscularis propria and is accompanied by moderate chronic edema, hemorrhage, fibrin deposition or urothelial changes; 3, severe inflammation in the lamina propria and muscularis propria accompanied by urothelial ulceration, severe chronic edema, hemorrhage and fibrin deposition).

### Real-time PCR analysis

mRNA expression levels were examined by quantitative real-time PCR (qRT-PCR). Total RNA was extracted from frozen bladder trigonum tissue specimens using TRIzol reagent (Invitrogen, Carlsbad, CA, USA), according to the manufacturer’s instructions. The total RNA was subsequently reverse transcribed into cDNA with a cDNA Synthesis Kit (TOYOBO, Shanghai, China). PCR was performed using RealMaster Mix with SYBR Green, which served as a fluorogenic reagent (TOYOBO, Shanghai, China). Each reaction comprised the following steps: 95 °C for 30 s, followed by 40 cycles of 95 °C for 5 s, 59 °C for 10 s, and 72 °C for 15 s. The expression level of each target gene was measured in triplicate in at least three independent experiments. The relative mRNA expression levels of the indicated genes were calculated using the ΔΔCT method and normalized to those of GAPDH, which was used as an endogenous control. The sequences of all the primers used for this experiment are listed in Table 3.

**Table 3:** Sequences of the PCR primers for the target genes.

Gene	forward	reverse	bp
CD44	CAGTCACAGACCTACCCAATTC	GTGTGTTCTATACTCGCCCTTC	101
IL-6	CTTCACAAGTCGGAGGCTTAAT	GCATCATCGCTGTTCATACAATC	103
STAT3	GGGCATCAATCCTGTGGTATAA	CAATCGGAGGCTTAGTGAAGAA	75
Bcl-xL	GGATACAGCTGGAGTCAGTTTAG	AGGATGGGTTGCCATTGAT	109
Mcl-1	TGCTTCGGAAACTGGACATTA	CCAGTTTGTTACGCCATCTTTG	92
HYAL-1	CTTCCCTGACTGCTACAACTAC	AAAGGGCATAGCTCTGGTTC	119
HYAL-2	GCCATCAGACCGAATAGTGAA	GCAGTCAGGAAAGAGGTAGAAG	136
GAPDH	TGCCAAGTATGATGACATCAAGAAG	AGCCCAGGATGCCCTTTAGT	71

### Immunoblotting

Total protein was extracted from the frozen bladder trigonum tissue specimens using lysis buffer (Beyotime, Shanghai, China), and the protein concentrations were determined using a BCA Assay Kit (Beyotime, Shanghai, China), according to the manufacturer’s instructions. The proteins (20–80 µg) were separated via SDS-PAGE and transferred onto PVDF membranes using a wet transfer apparatus (Bio-Rad, Hercules, CA, USA). The membranes were then blocked in 5% non-fat milk in TBST (0.1% Tween20 in Tris-buffered saline) before being incubated with anti-HYAL-1 (1:1000), anti-HYAL-2 (1:1000), anti-CD44 (diluted 1:1000), anti-IL6 (1:1000), anti-p-Stat3 (1:1000), anti-Stat3 (1:1000), anti-p-JAK2 (1:1000), anti-JAK2 (1:1000), anti-p-p65 (1:1000), anti-p65 (1:1000), anti-Bcl-xL (1:1000), anti-Mcl-1 (1:1000), and anti-GAPDH (1:1000) antibodies overnight at 4 °C. The membranes were subsequently incubated with the appropriate secondary antibodies (1:5000) for 2 h at room temperature, after which the protein bands were visualized by enhanced chemiluminescence (Millipore, Massachusetts, USA) and detected using an ImageQuant LAS4000 Mini Chemiluminescence Reader (GE, Fairfield, Connecticut USA). Protein expression was analyzed with ImageJ software.

### Immunohistochemical analysis

Five-micrometer-thick serial sections of bladder mucosal tissues were incubated with the appropriate primary antibodies overnight at 4 °C before being incubated with the appropriate secondary antibodies for 25 min at room temperature. The sections were then treated with peroxidase-marked streptavidin/peroxidase before being examined under an Olympus microscope (model BX-51, Japan). We used a semi-quantitative scoring system^51^ to grade the intensity of the immunoreactions. The positively stained cells in each bladder specimen were scored according to their staining intensity, which was graded using the following scale: 0 (no staining), +1 (weak but detectable staining), +2 (moderate staining) and +3 (intense staining). Five areas in each slide were evaluated under a microscope at a magnification of 40×. We calculated the H-score of each tissue sample by multiplying the percentage of cells in each intensity category by the corresponding staining intensity score and then adding these products together. The calculation was performed using the following formula: H-score = ∑(Pc × s), where s represents the intensity score, and Pc is the corresponding cell percentage.

### Statistical analysis

All data are presented as the mean ± standard deviation unless otherwise stated. Statistical analysis was performed by a blinded investigator using SPSS 19.0. The data were analyzed with Student’s tests. Associations with IL-6 and p-Stat3 were tested using nonparametric tests (Spearman correlation coefficient). P < 0.05 was considered statistically significant. All experiments were repeated independently at least three times.

### Data available statements

The datasets generated and analysed during the current study are not publicly available due to personal health information and exposure of his private parts, but are available from the corresponding author on reasonable request.

## Conclusion

This clinical study showed that intravesicular SH treatment had a significant effect on CCEG patients and that the IL-6/JAK2/Stat3 pathway is involved in the pathogenesis of CCEG. The animal experimental findings showed that treatment with SH decreased hyaluronidase expression, inhibited endogenous HA degradation, and reduced the interactions between HA degradation products and CD44 and thus suppressed CD44-dependent IL-6/JAK2/Stat3 pathway activation and downstream anti-apoptotic protein expression, namely, Mcl-1 and Bcl-xL expression. Therefore, intravesicular SH treatment may serve as an effective therapy for CCEG by inhibiting inflammation and proliferation.
